# Carbon-Supported Trimetallic Catalysts (PdAuNi/C) for Borohydride Oxidation Reaction

**DOI:** 10.3390/nano11061441

**Published:** 2021-05-29

**Authors:** Ahmed M. A. ElSheikh, Gordana Backović, Raisa C. P. Oliveira, César A. C. Sequeira, James McGregor, Biljana Šljukić, Diogo M. F. Santos

**Affiliations:** 1 Mechanical Engineering Department, South Valley University, AlShobaan AlMoslemeen Street, Qena 83521, Egypt; ahmed.elsheikh@eng.svu.edu.eg; 2 Department of Chemical and Biological Engineering, University of Sheffield, Sheffield S1 3JD, UK; james.mcgregor@sheffield.ac.uk; 3 Center of Physics and Engineering of Advanced Materials (CeFEMA), Instituto Superior Técnico, Universidade de Lisboa, 1049-001 Lisboa, Portugal; gordanabackovic@tecnico.ulisboa.pt (G.B.); raisa.oliveira@tecnico.ulisboa.pt (R.C.P.O.); cesarsequeira@tecnico.ulisboa.pt (C.A.C.S.); biljana.paunkovic@tecnico.ulisboa.pt (B.Š.)

**Keywords:** palladium, trimetallic catalysts, nanoparticle, borohydride oxidation, direct borohydride peroxide fuel cell, kinetic parameters

## Abstract

The synthesis of palladium-based trimetallic catalysts via a facile and scalable synthesis procedure was shown to yield highly promising materials for borohydride-based fuel cells, which are attractive for use in compact environments. This, thereby, provides a route to more environmentally friendly energy storage and generation systems. Carbon-supported trimetallic catalysts were herein prepared by three different routes: using a NaBH_4_-ethylene glycol complex (PdAuNi/C_SBEG_), a NaBH_4_-2-propanol complex (PdAuNi/C_SBIPA_), and a three-step route (PdAuNi/C_3-step_). Notably, PdAuNi/C_SBIPA_ yielded highly dispersed trimetallic alloy particles, as determined by XRD, EDX, ICP-OES, XPS, and TEM. The activity of the catalysts for borohydride oxidation reaction was assessed by cyclic voltammetry and RDE-based procedures, with results referenced to a Pd/C catalyst. A number of exchanged electrons close to eight was obtained for PdAuNi/C_3-step_ and PdAuNi/C_SBIPA_ (7.4 and 7.1, respectively), while the others, PdAuNi/C_SBEG_ and Pd/C_SBIPA_, presented lower values, 2.8 and 1.2, respectively. A direct borohydride-peroxide fuel cell employing PdAuNi/C_SBIPA_ catalyst in the anode attained a power density of 47.5 mW cm^−2^ at room temperature, while the elevation of temperature to 75 °C led to an approximately four-fold increase in power density to 175 mW cm^−2^. Trimetallic catalysts prepared via this synthesis route have significant potential for future development.

## 1. Introduction

Fuel cells (FCs) are electrochemical energy conversion devices with superior performance compared to conventional internal combustion engines, as they are not limited by the Carnot cycle [1]. Additionally, FCs are more environmentally friendly than conventional energy generation processes since they generally do not generate carbon dioxide (with few exceptions, such as direct alcohol/formic acid FCs) and do not rely on the consumption of fossil resources. Consequently, FC research and development has grown linearly with the worldwide energy demand and is projected to increase as FCs replace conventional heat engines in various applications [2]. However, many challenges need to be overcome if mass uptake of fuel cells is to be realized, including issues of safety, fuel source, and ensuring high efficiencies at low operating temperatures.

Low-temperature FCs targeted for use in portable applications and, particularly, for use in the transportation area have recently undergone rapid development [3]. They are compact, lightweight, and can attain high power densities. Polymer electrolyte membrane fuel cell (PEMFC), the most developed FC, most commonly comprises an H_2_-supplied anode and an O_2_-supplied cathode separated by a polymeric membrane. Nevertheless, employing H_2_ as fuel presents several challenges to widespread adoption, including safety, storage, and supply. Furthermore, H_2_-stream pretreatment is usually required in order to remove trace carbon monoxide, which poisons the electrode. To address these issues, H_2_/O_2_ PEMFCs can be replaced with direct liquid fuel cells (DLFCs). DLFCs can use organic and inorganic liquid fuels, which usually incorporate a supporting electrolyte in their composition [4].

Solutions of complex hydrides (e.g., LiBH_4_, NaBH_4_, or KBH_4_) are examples of hydrogen-containing compounds that can be used as fuels in DLFCs. Sodium borohydride (NaBH_4_) presents many advantages over other fuels. The open-circuit voltage (OCV) for the direct borohydride fuel cell (DBFC, 1.64 V) is higher than that of the direct methanol fuel cell (DMFC, 1.21 V) and the H_2_/O_2_ PEMFC (1.23 V). While the oxidation of H_2_ and methanol generates two and six electrons, respectively, per molecule, BH_4_^−^ direct oxidation generates eight electrons. Consequently, DBFC possesses specific energy over 50% higher than that of the DMFC [5]. Indig and Snyder [6] demonstrated in the 1960s that electricity could be produced via anodic oxidation of borohydride and O_2_ cathodic reduction, as shown in Equation (1) and Equation (2), respectively, to yield the overall fuel cell reaction (Equation (3)), where SHE stands for standard hydrogen electrode.

BOR NaBH_4_ + 8 OH^−^ → NaBO_2_ + 6 H_2_O + 8 e^−^   E^0^ = −1.24 vs. SHE (1)

ORR 2 O_2_ + 4 H_2_O + 8 e^−^ → 8 OH^−^   E^0^ = 0.40 vs. SHE (2)

Overall NaBH_4_ + 2 O_2_ → NaBO_2_ + 2 H_2_O   E_0_ = 1.64 V (3)

Many authors have discussed the advantages, disadvantages, and state-of-the-art in DBFCs, including postulating the replacement of O_2_ by hydrogen peroxide (H_2_O_2_) [7,8,9,10]. The use of H_2_O_2_ as an oxidant has become more popular in recent years, in particular as this results in an entirely liquid FC, thereby facilitating its use in applications where a compact design is required. When H_2_O_2_ is used as the oxidant, the catholyte consists of an acidic solution of H_2_O_2_ in order to avoid its decomposition into H_2_O and O_2_. Consequently, the DBFC in which the hydrogen peroxide reduction reaction (HPRR) takes place at the cathode and the BOR at the anode is known as the direct borohydride-peroxide fuel cell (DBPFC). It can generate electricity through Equations (1) and (4), with Equation (5) being the overall FC reaction.

HPRR H_2_O_2_ + 2e^−^ + 2H^+^ → 2 H_2_O   E^0^ = 1.78 V vs. SHE (4)

Overall NaBH_4_ + 4H_2_O_2_ → NaBO_2_ + 6H_2_O   E^0^ = 3.01 V vs. SHE (5)

The majority of published research on DBFCs discusses the need for effective and low-cost anode and cathode materials in order to facilitate the commercialization of DBFCs. This is a result of the high operating costs of the DBFC, which is correlated with the performance, efficiency, and cost of the electrocatalysts [5,8,9,10,11]. In fact, anodic catalysts for DBFCs must have high catalytic activity for BOR and be inert towards BH_4_^−^ hydrolysis, a chemical side reaction where BH_4_^−^ is consumed, generating H_2_ and BO_2_^−^. In addition, DBFC anodes need to exhibit chemical stability in a basic solution. Common anodic catalysts for DBFCs include noble metal catalysts such as Au [12,13,14,15,16], Pt [17,18,19,20], and their corresponding alloy materials [21,22,23,24], as well as some nonprecious metal catalysts, e.g., Ni [25,26], Zn [27], and Co [28,29,30]. In particular, Au has been extensively studied, as it was originally thought to be capable of inhibiting borohydride hydrolysis; however, it exhibits rather slow kinetics for borohydride oxidation [31,32]. Studies involving Au, Pd, and Pt bulk electrodes for BOR revealed that surface poisoning by reaction intermediates, including BH_ads_ or BH_3_, was significantly different for the three noble metals. Results have shown that this effect was more severe for Pt and also that the Au surface was the least affected by poisoning [33]. Despite this observation, Pt-based catalysts typically demonstrate faster electrode kinetics in BOR [24,34,35]; however, limited resources hinder their application as DBFC anodic catalysts. Pd is a promising alternative to Pt, presenting similar chemical properties and reactivity. Pd is considerably more abundant in the Earth’s crust than Pt (ca. fifty times) and hence presents fewer challenges regarding resource scarcity [36,37,38]. Therefore, Pd has been tested in FCs both as a cocatalyst with Pt and as a Pt-free catalyst [37,39,40,41]. Decreasing the required quantity of Pd by mixing it with non-noble metals in a well-designed catalytic structure capable of efficient borohydride oxidation can decrease the cost of membrane electrode assemblies (MEAs) and boost the commercialization of DBFCs. It was previously found that co-doping can improve the catalytic performance of noble metal catalysts [42,43,44]. For example, a Pd-Co/C nanocatalyst has been demonstrated as an electrocatalyst for formic acid oxidation and methanol oxidation [45,46]. 

Previous studies reported Pd nanocatalysts and Pd nanoalloys as anodic catalysts for BOR in DBFCs [47,48]. Furthermore, Simões et al. [49] analyzed how Pd, Au, and Pd-Au carbon-supported nanoparticles with different compositions performed during BOR in alkaline media. Pd_x_Au_1-x_/C electrocatalyst exhibited an open circuit potential (OCP) lower than Pd and Au did. Merino-Jimenez et al. [50] evaluated a titanium-supported and carbon nanofiber-covered Pd-Ir alloy for BOR. The authors concluded that alloying Pd with Ir improves Pd electroactivity towards BOR. This enhancement was associated with the favoring of BOR (direct oxidation) and suppression of hydrogen evolution reaction (HER). Furthermore, Grimmer et al. [51] studied BOR at Pd/C by cyclic voltammetry, rotating disc electrode (RDE) measurements, chronoamperometry coupled to NMR spectrometry, and in situ Fourier transform infrared spectroscopy (FTIR). RDE results yielded n = 4 (at 0.4 V vs. reversible hydrogen electrode (RHE)) and n = 8 (at 0.8 V vs. RHE). Thus, at 0.8 V vs. RHE, BOR is 100% direct, while at 0.4 V, two different pathways are proposed to operate: (i) the total oxidation of BH_4_^−^ to BO_2_^−^ and two H_2_ molecules and (ii) a four-electron electrooxidation with the production of BH_2_^−^ species. The authors also suggested that at low potentials, Pd-BH_2_OH species block surface sites, while at higher potentials, this intermediate is fully oxidized (n = 8). Additionally, Pd has been shown to be relatively unharmed by surface poisoning, preserving good BOR kinetics even in highly concentrated BH_4_^−^ solutions relevant for DBPFC operation [33]. Recently, by screening selected metal combinations (e.g., Pd with Co, Au, Ti) over a wide range of compositions, bimetallic and trimetallic Pd-based materials with optimum compositions that exhibited good activity for BOR were identified [51,52,53,54,55,56]. According to multiple published reports, Ni presents several benefits when added as a cocatalyst to C-supported Pd. First, it is an Earth-abundant metal, and therefore its use tackles issues of resource scarcity and reduces cost. Secondly, it is oxyphilic and capable of generating the oxygen species necessary for the redox reaction. Finally, the presence of Ni can exert an electronic effect over Pd, altering and potentially enhancing the catalytic properties of the latter [57,58,59].

In this work, investigations on the oxidation of BH_4_^−^ in alkaline media at different working conditions are presented for a selection of trimetallic carbon-supported catalysts based on Pd (PdAuNi/C), prepared by a facile and scalable synthetic route. There is a consensus that adding a second metal as a cocatalyst to Pd is beneficial for its electrocatalytic performance. This is due to the electronic, geometric, and bifunctional effects that the second metal can provide. Previous works have considered multiplying the catalytic benefits by adding two metals to Pd instead of one [56,60,61,62,63,64,65,66]. It is hypothesized that adding two metals, such as Au and Ni, each of which is known to be a beneficial addition to Pd individually [67,68,69], would significantly enhance Pd electrocatalytic performance. A small number of previous studies have previously investigated PdAuNi electrocatalysts [67,68,69,70,71,72]. Au [12,73,74,75,76,77] and Ni [25,78,79,80], like Pd, have been proven to possess good catalytic activity towards BOR, which makes their incorporation in a catalyst additionally attractive. Specifically, in the case of Ni electrodes, the formation of β-NiOOH has been pointed out as essential for the oxidation of BH_4_^−^ [26]. Furthermore, to maximize the potential for BOR, a high-surface inert, mesoporous, and conductive support material is necessary to anchor the trimetallic nanoparticles and prevent their coalescence. Vulcan carbon (XC-72R) is commonly applied for this application due to its high surface area, mesoporous structure, and affordable cost. It was therefore chosen to support the PdAuNi and Pd nanoparticles. There are various chemical and physical synthetic routes in the literature to prepare dispersed metal nanoparticles [81]. The synthesis protocol is thought to play a crucial role and contribute to the catalyst physicochemical and catalytic characteristics. Any chosen method should balance between fine-tuned catalytic properties and ease of preparation, leading potentially to facile mass production [82]. NaBH_4_ metal reduction is a very common means to prepare C-supported catalysts due to its easy procedure and quality-produced catalysts. Moreover, there is more than one NaBH_4_ protocol to prepare Pd-supported nanoparticles. This work aims to synthesize three carbon-based trimetallic catalysts based on different borohydride reduction protocols. The physicochemical and morphological properties of those materials were determined and compared. Their performance as electrocatalysts for BOR was analyzed and discussed considering the different synthesis routes employed and the final properties obtained for each material. 

## 2. Materials and Methods

### 2.1. Chemicals

Metallic precursors (PdCl_2_, NiCl_2_, and AuCl_3_) to synthesize PdAuNi/C electrocatalysts were purchased from Sigma-Aldrich (St. Louis, MO, USA). Vulcan carbon (XC-72R), purchased from Cabot Corp (Boston, MA, USA), was used to support the synthesized metal nanoparticles. NaBH_4_ (SB, 96 wt.%), ethylene glycol (EG, 99.5 wt.%), 2-propanol (IPA, 99 wt.%), NaOH (85 wt.%), and ethanol (100%) were purchased from Sigma-Aldrich. For the electrochemical measurements, NaBH_4_ (98 wt.%, Scharlau, Barcelona, Spain) and H_2_O_2_ (30 vol.%, Carlo Erba, Barcelona, Spain) were used. All chemicals were used as received without further purification.

### 2.2. Catalyst Preparation

Three borohydride reduction protocols using (i) a NaBH_4_-ethylene glycol complex, (ii) a NaBH_4_-2-propanol complex, and (iii) a three-step route were deployed to synthesize carbon-supported PdAuNi catalysts denoted herein as PdAuNi/C_SBEG_, PdAuNi/C_SBIPA_, and PdAuNi/C_3-step_, respectively. The theoretical metal loading based on the quantity of metal salts used in the synthesis was 12 wt.% for PdAuNi/C_SBIPA_ and 20 wt.% for both PdAuNi/C_SBEG_ and PdAuNi/C_3-step_. Although electrocatalysts for laboratory studies usually load the carbon support with 20 to 40 wt.% of metal, the use of lower loadings is more representative of industrial catalyst loadings which are typically ~5 wt.% [83]. 

The SBEG synthesis protocol uses a NaBH_4_-ethylene glycol complex, following previous work [36,84,85]. Vulcan carbon (202 mg) was first dispersed in ethylene glycol (10 mL). The metallic precursor solution containing PdCl_2_ (28 mg mL^−1^), AuCl_3_ (55 mg mL^−1^), and NiCl_2_ (36.7 mg mL^−1^) was then suspended in ethylene glycol (10 mL). The two suspensions were subsequently mixed. Next, a solution of NaBH_4_ (20 mL) was slowly added to the metal and carbon mixture under magnetic stirring. The mixture was heated to 35 °C and kept at that temperature for 3.5 h. The mixture was then left to cool and washed copiously with deionized water until neutralization. Following this, it was dried in a vacuum oven at 80 °C for 2 h.

The second reduction protocol (SBIPA) applied NaBH_4_ following methods described in previous reports [86,87,88,89]. The Vulcan carbon and metal precursors were sonicated in a mixture of 2-propanol/water (50 vol.%). KBr was added to stabilize the metal nanoparticles. The mechanism of this is that the larger Br^−^ ions substitute for Cl^−^ ions in solution, stabilizing Pd particles and preventing further particle agglomeration [90]. Unlike an organic surfactant such as PVP, KBr can be washed off easily after synthesis from the particle surface. The KBr/metal atomic ratio was 1.5. The mixture was stirred for 10 min, followed by the addition of NaBH_4_ solution (0.5 M, 15 mL). Stirring then continued for another 30 min. Following this, the sample was washed under vacuum filtration. Finally, the wet powder was dried at 80 °C in a vacuum oven overnight. 

The third protocol to prepare PdAuNi/C (three-step) aimed to compare the stepwise reduction of metals on the carbon surface to the co-reduction procedure practiced in the first two protocols. Consecutive reduction of different metals can lead to the formation of core@shell structures, such as Ru@Pd/C [91], Au@Pd [92,93], and Cu@Pd [94]. The NaBH_4_-2-propanol reduction mixture was once more used to prepare this catalyst. The procedure started with sonicating Vulcan carbon (202.4 mg) in a mixture of 2-propanol/water (50 vol.%). Then, NiCl_2_ was added and kept in the solution under stirring for 1 h. This was longer than the reduction time required for Au and Pd reduction time, as Ni is more oxyphilic and less reducible [95,96]. Subsequently, AuCl_3_ was added with stirring for 30 min. Finally, PdCl_2_ was added with stirring for 30 min. To illustrate the impact of adding both Au and Ni onto Pd, a monometallic C-supported Pd catalyst (Pd/C_SBIPA_) was synthesized using the SBIPA procedure.

### 2.3. Physical Characterization

The crystal structure of the prepared catalysts was investigated using X-ray diffraction (XRD) with a Bruker D2 Phaser operating using Cu K_α_ radiation at 30 kV, 10 mA, and 12° min^−1^ scan rate. To evaluate the crystallite size, the Scherrer equation was applied using the Pd (111) peak details. To quantify the metallic species in each catalyst powder, inductively coupled plasma optical emission spectrometry (ICP-OES) was applied using a Spectro Ciros Vision spectrometer by Spectro Analytical Instruments Inc. (Kleve, Germany). The samples were first digested in HNO_3_ (2%), followed by 10-fold dilution in aqua-regia (8%). The surface-weighted chemical composition of the catalysts was examined through energy-dispersive X-ray spectroscopy (EDX) attached to a JEOL JSM 6010LA scanning electron microscope (SEM) by JEOL Ltd. (Tokyo, Japan). Each catalyst surface was examined twice applying two different accelerating voltages: 10 kV and 20 kV [97] enabling studying the composition at two different depths. The catalyst morphology was examined by transmission electron microscopy (TEM) employing a Phillips C100 microscope (Hillsboro, OR, USA) operating at 100 kV applying LaB_6_ filament. The TEM samples were prepared by suspending 5 mg of each sample powder in 2 mL of ethanol. Then, the mixture was sonicated for 1 h. A spray atomizer was then used to take 10 µL of the suspension to paint the holey carbon-coated Cu grid. The grid was left to dry for 24 h. The surface chemistry of the prepared electrocatalysts was studied by X-ray photoelectron spectroscopy (XPS) employing a Thermo Scientific K-Alpha+ spectrometer (Waltham, MA, USA). An Al-X ray source (72 W) and 400-μm^2^ were applied. The pass energy to record the data was 150 eV for survey scans and 40 eV for high-resolution ones. The survey scan step size was 1 eV, but that of the high-resolution was 0.1 eV. Low-energy electrons and argon ions were used to neutralize the charge. CasaXPS from Casa Software Ltd. (Teignmouth, UK) was used to analyze the data and employed a Shirley-type background and Scofield cross-sections with an energy dependence of −0.6.

### 2.4. Working Electrode Preparation

Catalytic inks were prepared by ultrasonically dispersing 5 mg of each catalyst in 2 mL of ethanol and 25 µL of 5 wt.% Nafion suspension for 30 min. Subsequently, 5 µL of the prepared ink was deposited onto a glassy carbon electrode (GC, A = 0.0707 cm^2^, inactive for BOR [98]) and left to dry at room temperature for 1 h. Catalyst loading was 0.175 mg cm^−2^ for all catalysts. The total metal loadings (calculated based on the ICP-OES results) were 0.021, 0.027, 0.039, and 0.015 mg cm^−2^ for Pd/C_SBIPA_, PdAuNi/C_SBEG_, PdAuNi/C_3-step_ and PdAuNi/C_SBIPA_, respectively. Pd loadings were 2.51, 2.41, 4.77, and 5.77 μg cm^−2^ for Pd/C_SBIPA_, PdAuNi/C_SBEG_, PdAuNi/C_3-step_, and PdAuNi/C_SBIPA_, respectively. 

### 2.5. Electrochemical Evaluation

Electrochemical characterization was carried out in a conventional three-electrode setup of 125 mL. An ALS 2325 bipotentiostat (ALS Co., Ltd, Japan) combined with an RRDE-3A apparatus was used for fundamental experiments. Pt served as a counter electrode, and a saturated calomel electrode (SCE) as a reference electrode. All potential values within this paper were converted and are presented relative to RHE.

BOR measurements were carried out using cyclic and linear scan voltammetry. A 2 M NaOH solution was employed as the supporting electrolyte for all fundamental experiments. Cyclic voltammetry (CV) measurements were carried out in the potential range from OCP to 1.2 V applying scan rates from 5 to 1000 mV s^−1^. Furthermore, the temperature effect was analyzed in the 25–65 °C temperature range. The BH_4_^−^ concentration influence was studied in the 0.01–0.12 M range. Studies on the effect of temperature and concentration were conducted at a scan rate of 50 mV s^−1^. RDE studies were carried out by linear scan voltammetry (LSV) at 10 mV s^−1^ by applying different rotation rates (0–2400 rpm). Apart from the concentration study, all experiments were conducted in a 0.03 M NaBH_4_ in 2 M NaOH solution. Experiments were run at 25 °C and 0 rpm unless otherwise stated.

### 2.6. Fuel Cell Testing

A laboratory-scale direct borohydride peroxide fuel cell (DBPFC, V = 100 mL for both anodic and cathodic compartments) was assembled and tested. The two compartments were separated by a Nafion^®^117 (DuPont, Wilmington, DE, USA) cation-exchange membrane. A PAR 273A (Princeton Applied Research, Inc., Oak Ridge, TN, USA) potentiostat using PowerSuite software (PowerCV+ PowerSTEP) was employed for control of the experiments. The anolyte and catholyte were 1 M NaBH_4_ in 4 M NaOH and 5 M H_2_O_2_ in 1.5 M HCl solutions, respectively. The chosen compositions are proven to be optimal in previous studies [99,100]. PdAuNi/C_SBIPA_ was employed as the anodic catalyst (1 cm^2^, 0.100 mg cm^−2^ catalyst loading, 0.012 mg cm^−2^ metal loading). The catalytic ink used for the anode was prepared as described in Section 2.4, with a GC tip used as substrate electrode. The cathode was a Pt mesh with a high surface area (Johnson Matthey, A = 50 cm^2^) to ensure that fuel cell performance was not limited by the cathodic process. Interelectrode distance was the lowest possible to minimize ohmic resistance. DBPFC performance was analyzed in the 25–65 °C temperature range. Cell polarization curves were recorded, and the corresponding power density curves were plotted.

## 3. Results and Discussion

### 3.1. Physical Characterization Results

**Figure 1:**
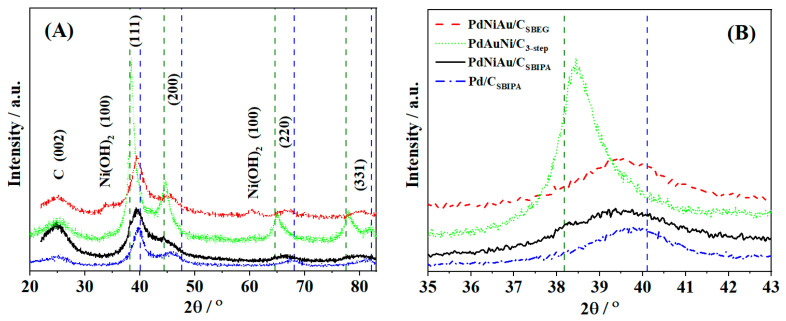
(**A**) XRD patterns of PdAuNi/C_SBEG_, PdAuNi/C_SBIPA_, Pd/C_SBIPA_, and PdAuNi/C_3-step_ and (**B**) the enlarged (111) facet for the four catalysts.

Figure 1 shows the XRD patterns of Pd/C and the three trimetallic catalysts. All catalysts showed one broad peak at 25°, typical of graphitic Vulcan carbon. For the monometallic Pd/C, the pattern showed four peaks that are characteristic of Pd (111), (200), (220), and (311) faces at 39.8°, 46.5°, 68.3°, and 82.1°, respectively (PDF#46-1043). However, for both trimetallic catalysts prepared by co-reduction (SBEG and SBIPA), there was a small shift in the peak positions to lower diffraction angles. This shift is ascribed to alloying among the three metals. Pd and Au, in particular, are well-known to exhibit remarkable alloying behavior [95,101,102,103,104,105,106,107,108]. Although Pd and Ni are known to form a solid solution for their bulk alloys, their potential for formulating a nanoalloy is lower [96,107,108,109]. It is not uncommon to see distinctive Ni(OH)_2_ peaks at 60.1°, which is indicative of bulk Ni species segregation. In the present study, the shift in the two co-reduced trimetallic samples was suggestive of a nanoalloy formation among the metals. However, the appearance of Ni(OH)_2_ peaks at 33.4° and 60.1° in the case of PdAuNi/C_SBEG_ implied there was some Ni segregation in the bulk of that catalyst. Additionally, these Ni(OH)_2_ peaks were also present in the three-step catalyst, although smaller than that of PdAuNi/C_SBEG_.

In contrast to PdAuN/C_3-step_ and PdAuNi/C_SBEG_, the catalyst synthesized via the SBIPA protocol did not show any individual Ni peaks. This indicated a higher potential of mixing of the three metals when the SBIPA protocol was used. KBr addition may also have contributed due to the replacement of the smaller Cl^−^ anion with Br^−^ during synthesis [110]. 

Unlike the SBEG and SBIPA catalysts, the XRD peaks of PdAuNi/C_3-step_ were closer to pure Au (PDF#04-0784) than pure Pd (PDF#46-1043). This suggested the formation of Au@Pd (core@shell) structure as a consequence of the stepwise reduction process as previously reported [92,93]. Another important observation is that the XRD peak shift to lower diffraction angles was suggestive of the expansion of the Pd crystal lattice by the incorporation of the larger Au atoms into the Pd lattice. Ni is, theoretically, expected to contract the lattice, not to expand it. However, previous studies have found that adding Ni to Pd does not seem to displace the Pd diffraction peaks and therefore Ni does not necessarily contract the Pd lattice [59,111,112]. The peak shift in the case of PdAuNi/C_SBEG_ and PdAuNi/C_SBIPA_ is therefore consistent with lattice expansion and tensile strain that could upshift the *d*-band center and decrease the adsorbate-Pd bond strength [105].

The XRD Pd(111) peak of the four catalysts was analyzed to extract the peak broadening and diffraction angle values of each sample, and subsequently to determine their structural parameters: particle size, interplanar distance, and lattice constants (Table 1). The detailed analysis is explained in section “1. XRD calculation” of supplementary information (SI). While the interplanar distance and lattice constant were the same for Pd/C_SBIPA_, PdAuNi/C_SBIPA_, PdAuNi/C_SBEG_ (2.26 and 3.92 Å, respectively), it has increased in the case of PdAuNi/C_3-step_ to 2.33 and 4.03 Å, respectively. It is noteworthy that the smallest particle size and highest broadening (FWHM) were those of PdAuNi/C_SBIPA_, followed by the monometallic Pd/C_SBIPA_, PdAuNi/C_SBEG_, and, finally, PdAuNi/C_3-step_. Factors other than crystallite size can also contribute to peak broadening, e.g., the instrument employed and the lattice strain. However, the Scherrer equation (Equation (S3)) is known to provide a reliable approximation of the crystallite size. The large particle size of PdAuNi/C_3-step_ could be attributed to its Au-like structure, which presents larger lattice and interplanar distances.

The bulk elemental composition of the synthesized catalysts was investigated by ICP-OES, as illustrated in Table 2. For Pd/C_SBIPA_, the identified Pd loading was 12 wt.%, equivalent to the theoretical value. For PdAuNi/C_SBIPA_, PdAuNi/C_SBEG_, and PdAuNi/C_3-step_, Pd loadings of 9 wt.%, 16 wt.%, and 22 wt.% were identified, respectively.

**Figure 2:**
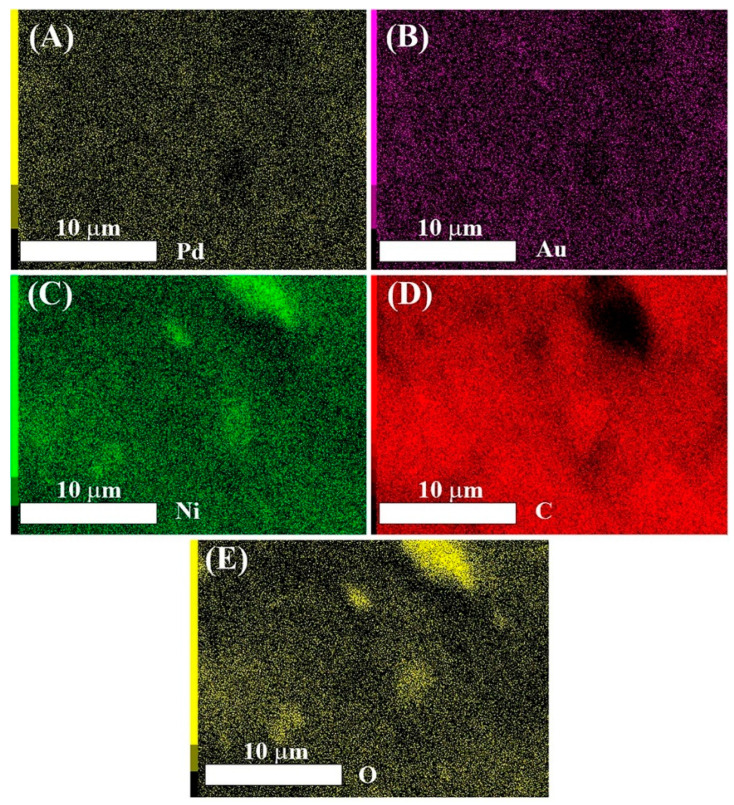
Elemental maps of (**A**) Pd, (**B**) Au, (**C**) Ni, (**D**) C, and (**E**) O obtained by EDX spectroscopy of PdAuNi/C_SBEG_ at 10 kV.

While ICP-OES determines the whole composition of the catalysts, the catalytically active sites are not dispersed throughout the bulk of the material. Instead, interaction with reactants takes place at surface sites. Therefore, the surface-weighted technique of EDX was used to gain insights into the composition of the catalysts in the catalytically relevant surface region. Figure 2 shows EDX metal elemental maps of PdAuNi/C_SBEG_. 

While the Pd (A) and Au (B) maps show similar and relatively even distribution of both metals, the Ni map (C) shows three areas that were more densely populated with Ni species, indicating that Ni was not homogeneously distributed at the surface. The regions of high Ni density correspond to regions of low density in the C map (D) and in the O map (E). This may suggest that some bulk segregation of Ni(OH)_2_ had occurred, as supported by XRD data where a Ni(OH)_2_ peak was observed, Figure 1.

Table 3 shows the quantitative composition data extracted from EDX in both weight and atomic percent for the three trimetallic catalysts investigated. This aimed to highlight the different surface metal segregation by applying two accelerating voltages: 10 kV and 20 kV, the latter providing greater surface penetration. Unlike Pd and Au, the atomic concentration of Ni at 10 kV was larger than that at 20 kV. This further confirms that Ni segregation to the catalyst surface occurred. EDX analyses revealed higher metal loading than either ICP-OES or the theoretical compositions, as the synthesis resulted in a heterogeneous composition throughout the carbon support, with metal preferentially located towards the surface, beneficial for catalysis. 

**Figure 3:**
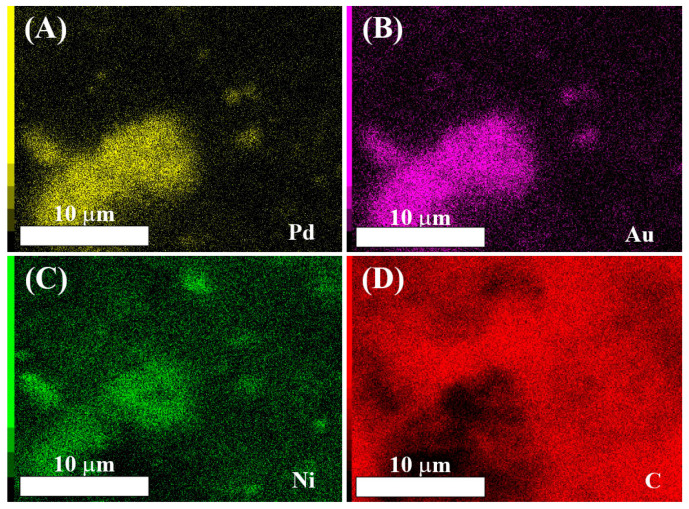
Elemental maps of (**A**) Pd, (**B**) Au, (**C**) Ni, and (**D**) C of PdAuNi/C_SBIPA_ obtained by EDX spectroscopy at 10 kV.

Figure 3 shows the EDX maps of PdAuNi/C_SBIPA_. Unlike PdAuNi/C_SBEG_, the Au (A), Pd (B), and Ni (C) maps show that the three metals were co-located with high-density regions of one metal corresponding to high-density regions of the others. Areas of low metal density corresponded to density regions in the C map (D). This is anticipated, as at regions of low metal density, the bare C support was exposed at the surface. Although to a lesser extent than PdAuNi/C_SBEG_, the quantitative data for PdAuNi/C_SBIPA_ (Table 3) indicated a slightly higher concentration of Ni at 10 kV (2.90 at.%) as compared to that at 20 kV (2.52 at.%), perhaps indicating some surface segregation of Ni for this material as well. Overall, PdAuNi/C_SBIPA_ showed relatively homogeneous metal distribution throughout the surface region, indicative of good mixing characteristics of the three metals in this case. This was further supported by the absence of any distinctive Ni peaks in this catalyst XRD pattern. Similar to PdAuNi/C_SBIPA_, the EDX analysis of PdAuNi/C_3-step_ revealed that Pd, Au, Ni, and C were co-located with each other (Figure S1), suggesting good mixing and little Ni segregation in this material.

**Figure 4:**
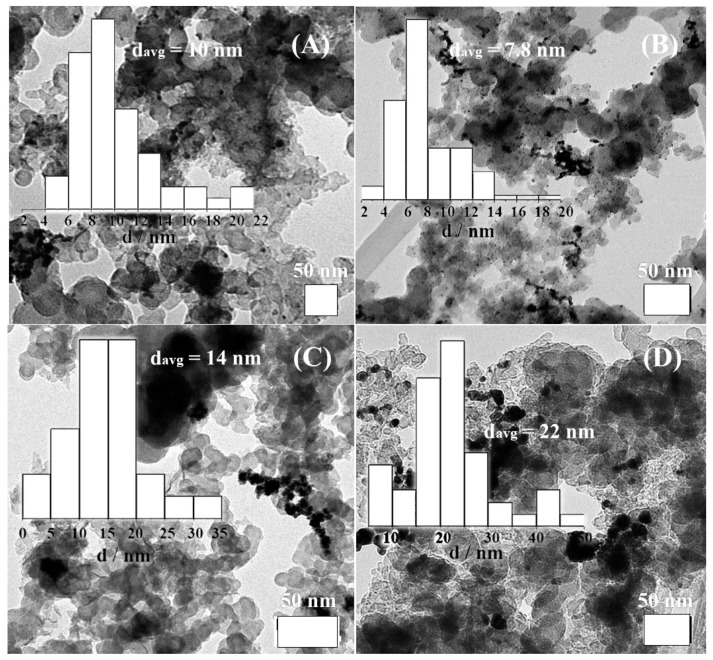
TEM micrographs and corresponding particle size distribution histograms of (**A**) Pd/C_SBIPA_, (**B**) PdAuNi/C_SBIPA_, (**C**) PdAuNi/C_SBEG_, and (**D**) PdAuNi/C_3-step_.

Figure 4 shows representative TEM micrographs obtained for the Pd/C_SBIPA_, PdAuNi/C_SBIPA_, PdAuNi/C_SBEG_, and PdAuNi/C_3-step_ electrocatalysts, with additional TEM images being included in SI (Figures S2–S5). Examining the micrographs, the presence of highly dispersed metal nanoparticles on the carbon surface could be inferred. The very small particles indicated metal nanoparticles, while the large grey particles (40–60 nm) indicated the carbon aggregates. However, some particle aggregation and agglomeration could also be seen. This could be due to the synthesis method and/or being a monometallic system, which can promote particle growth and agglomeration more than a bimetallic or trimetallic one [56]. Comparing the trimetallic PdAuNi/C_SBIPA_ catalyst with the monometallic one prepared by applying the same protocol suggests that the latter explanation may apply in this case.

**Figure 5:**
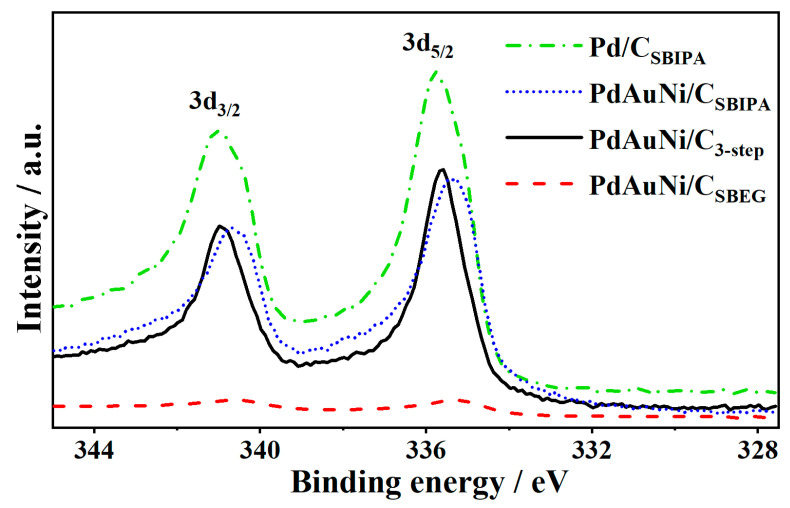
XPS spectra of Pd 3d in PdAuNi/C_SBIPA_, Pd/C_SBIPA_, PdAuNi/C_SBEG_, and PdAuNi/C_3-step_, showing Pd 3d_3/2_ and Pd 3d_5/2_ peaks.

PdAuNi/C_SBIPA_ particles (1–6 nm, consistent with XRD analysis, Table 1) were very well dispersed, and less particle aggregation could be seen as compared to Pd/C. The reverse situation was noted for the PdAuNi/C_SBEG_, which showed significant particle aggregation. Above those few carbon particles, high metal particle aggregation could be seen. This could be promoted by the NaBH_4_-ethylene glycol reduction complex at 40 °C. Additionally, as discussed above with reference to the EDX map (Figure 3C), the high Ni content in this catalyst might contribute to this agglomeration, noting that Ni exists only in the form of Ni(OH)_2_ (Figure 5).

The preparation method may also play a role in the promotion of agglomeration. For PdAuNi/C_SBEG_, the metal precursors were initially mixed in ethylene glycol, followed by the slow addition of NaBH_4_. Once the NaBH_4_ addition commenced, the orange/brown solution turned black instantly, alongside the formation of bubbles. PdAuNi particles had most likely been reduced and segregated before the carbon support was added. For PdAuNi/C_3-step_, on the other hand, Ni was initially reduced on the carbon surface (for 30 min), followed by Au (for 30 min), and finally Pd (for 30 min). As is clear from the XRD and EDX measurements, Ni segregation occurred once again, further verified by the observation of Ni(OH)_2_ peaks, albeit in lower intensity than for PdAuNi/C_SBEG_. Additionally, Au had most likely formed a core@shell structure with Pd, since the XRD pattern was more representative of Au than of Pd. Although to a lesser degree than PdAuNi/C_SBEG_, significant particle aggregation was observed, and particle size was the largest in comparison to the other catalysts. Following PdAuNi/C_3-step_, PdAuNi/C_SBEG_ particle size was larger than that of both Pd/C_SBIPA_ and PdAuNi/C_SBIPA_. The PdAuNi particle sizes obtained were slightly smaller than previously reported for trimetallic systems [68,69].

Figure 5 shows the XPS measured data for the Pd 3d peak positions of the four catalysts. The molar Pd:Au:Ni ratio of PdAuNi/C_SBIPA_ equaled 64:17:19, while it was 5:1:94 and 58:6:36 for PdAuNi/C_SBEG_ and PdAuNi/C_3-step_, respectively. This showed the abundant Pd presence on the surface of PdAuNi/C_3-step_ and PdAuNi/C_SBIPA_ samples, while PdAuNi/C_SBEG_ contained mainly Ni (94 at.%) and only 6 at.% Pd and Au combined. Furthermore, it is noteworthy that the highest oxygen content (5.19 at.%) existed in PdAuNi/C_SBEG_, likely a consequence of the presence of significant Ni(OH)_2_ (2.19 at.%). The Pd 3d peak of Pd/C_SBIPA_ (Table S1 in SI) could be deconvoluted into a high-energy band corresponding to Pd 3d_3/2_ (340.73 eV) and a low-energy band corresponding to Pd 3d_5/2_ (335.43 eV). Those bands were shifted approximately 0.40 eV to higher binding energy compared to pure Pd, due to the interaction with the C support. Moreover, the Pd 3d binding energies of PdAuNi/C_SBEG_ and PdAuNi/C_SBIPA_ samples were shifted ~0.5 eV and 0.15 eV, respectively, to lower binding energies. On the contrary, Pd 3d of PdAuNi/C_3-step_ was shifted 0.05 eV to binding energy higher than that of the Pd/C_SBIPA_ sample. Pd 3d peak positions are shown in Figure 5. Ni 2p and Au 4f were also shifted in the three trimetallic samples compared to pure Au and Ni, the binding energies of which were 84 eV and 855.6 eV (Ni(OH)_2_), respectively. While the Ni peaks shifted towards higher binding energy compared to pure Ni, the reverse trend was observed for Au. The Ni 2p shift was estimated to be +0.3 eV, +0.7 eV, and +0.8 eV for PdAuNi/C_SBIPA_, PdAuNi/C_SBEG_, and PdAuNi/C_3-step_, respectively. The Au 4f shift was −0.3 eV, −0.4 eV, and −0.2 eV for PdAuNi/C_SBIPA_, PdAuNi/C_SBEG_, and PdAuNi/C_3-step_, respectively. The shift in the binding energy was indicative of a change in the adsorption behavior of the respective catalysts. The presence of Au and Ni potentially explains why PdO was not detected in the trimetallic sample, unlike in the monometallic one, which contained 20 wt.% of Pd as PdO. Adding both Au and Ni enhanced Pd air stability. The full XPS surveys are illustrated in Figure S6. The individual elemental (Pd 3d, Au 4f, and Ni 2p) XPS peaks are available in Table S1, which confirms that Pd and Au existed in the metallic state in the trimetallic samples, Pd existed in metallic and oxide form in the monometallic sample, and Ni predominantly existed as Ni(OH)_2_. The Ni 2p peak in the PdAuNi/C_SBIPA_ sample contained some Ni metal alongside Ni(OH)_2_, unlike PdAuNi/C_SBEG_ and PdAuNi/C_3-step_. This further exemplifies the good mixing of the three metals obtained by the SBIPA protocol, which was corroborated by the XRD and EDX data.

PdAuNi/C_SBEG_ has furthermore achieved the highest shift of Pd 3d to the left, which was also the broadest from the four-sample Pd 3d peaks as reported for the addition of Ni to Pd [108]. Examining the Ni 2p peaks of the three trimetallic samples, two high-binding-energy satellite peaks were adjoined to the main peaks of Ni, which indicates Ni multi-electron excitations [59,96,111,112]. Additionally, in the case of PdAuNi/C_SBEG_ and PdAuNi/C_3-step_ samples, the single pattern of two Ni 2p peaks suggests the presence of only one oxide species, Ni(OH)_2_, in contrast to the findings of Ramulifho et al. [113]. In terms of co-reduction, Au is the easiest of the three metals to reduce, followed by Pd. On the contrary, Ni is the slowest to reduce [95,114]. Therefore, it may not be incorrect to assume that the surface of the trimetallic catalysts (especially PdAuNi/C_SBEG_) was richest in Ni and poor in Au, which was evident from the XPS and EDX measurements. On the other hand, the core would be highly populated with Au and lacking a substantial Ni proportion.

### 3.2. Borohydride Oxidation Reaction Studies

**Figure 6:**
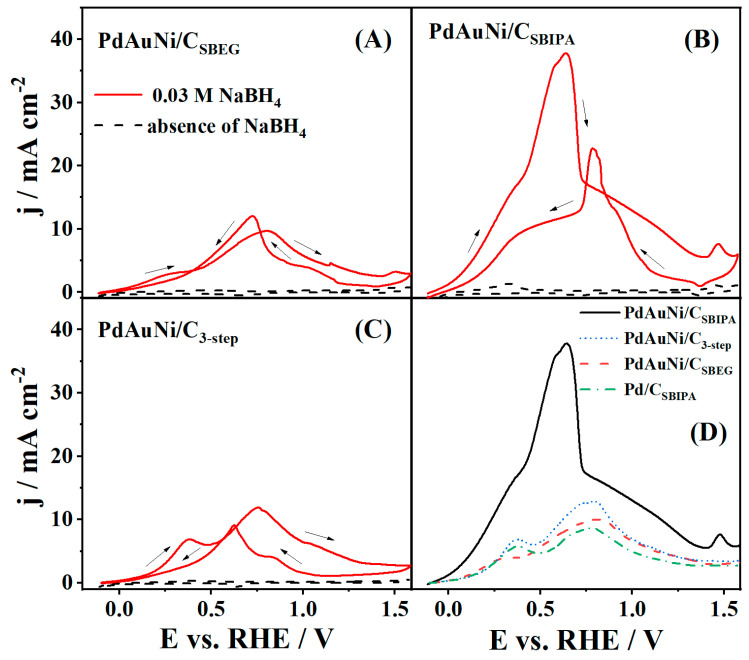
CVs (third cycle) in the absence (2 M NaOH) and presence (2 M NaOH + 0.03 M NaBH_4_) of BH_4_^−^ for (**A**) PdAuNi/C_SBEG_, (**B**) PdAuNi/C_SBIPA_, and (**C**) PdAuNi/C_3-step_ electrocatalysts, and (**D**) comparative CVs of trimetallic and Pd/C_SBIPA_ monometallic catalysts in the presence of BH_4_^−^. CVs ran at 50 mV s^−1^ and 25 °C.

The catalytic performance of the three PdAuNi/C electrocatalysts towards the BOR was initially investigated by scanning comparative CVs in 2 M NaOH with 0.03 M NaBH_4_ and in pure 2 M NaOH. Along with evaluating the activity of the synthesized trimetallic catalysts, the response of monometallic Pd/C was also evaluated in order to better understand the role played by Au and Ni. The CVs of three catalysts as well as of monometallic Pd/C depicted in Figure 6A–C show the absence of any relevant anodic peaks in the BOR potential region in NaOH solution, implying that the current generated during CVs recorded in the presence of BH_4_^−^ originated from its oxidation. Based on the shape of the CVs and similar OCP values of ca. −0.12 V, it could be assumed that the studied catalysts promoted similar reaction mechanisms. Similar OCP values were reported in other recent studies on BOR at Pd-containing materials [53,54]. Within the scanned potential range, CVs of three different catalysts showed two oxidation peaks in the positive scan, while an additional sharp oxidation peak was evident in the reverse scan. The first oxidation peak appeared as a wide oxidation hump at ca. 0.29 V for the PdAuNi/C_SBEG_ and PdAuNi/C_SBIPA_ catalysts, while in the case of PdAuNi/C_3-step_, this peak was more pronounced and positioned at more positive potential (0.38 V). It is believed that this peak can be attributed to H_2_ oxidation, which originates from the borohydride hydrolysis reaction, and to the initial oxidation of adsorbed BH_3_OH^−^ [54]. The second anodic peak at ~0.8 V for all examined samples can be considered to originate from electrooxidation of BH_4_^−^ as well as BH_3_OH^−^ [48,54]. PdAuNi/C_SBIPA_ gave the best catalytic response, reaching the peak current density of 46 mA cm^−2^ at 50 mV s^−1^, a value considerably higher than those reached employing carbon-supported Pd-Cu [115], Pd-Au [116], and Au-Ni [117] alloy nanoparticles. Additionally, the oxidation peak on the cathodic scan in the potential window from 0.5 to 0.8 V for all three studied samples was most likely related to the oxidation of BH_3_OH^−^ [118], which was formed during the anodic scan and stayed adsorbed on the oxidized electrode surface until it was reactivated by reduction of the surface oxides. 

The catalytic performance of the synthesized materials was further compared with the response obtained for monometallic Pd/C_SBIPA_. Figure 6D reveals the inferior catalytic activity of Pd/C_SBIPA_. Introducing Au and Ni into Pd provided more active sites where BH_4_^−^ adsorption could take place and as such, trimetallic catalysts were expected to exhibit higher activity towards BOR. Improved catalytic properties can be explained by the produced synergistic effect of combining Pd with Au, a metal that is relatively inert towards hydrolysis, and Ni, a 3d transition metal, and the resultant electronic effect. In terms of electronic interaction, as XPS results suggest, the addition of Ni may have influenced the atomic band structure of the other two metals. This can further lead to the reduced adsorption energy of BOR intermediates, thus preserving those active sites for BH_4_^−^ adsorption and further encouraging BOR activity [119]. 

The PdAuNi/C_SBIPA_ highest catalytic activity most likely resulted from the absence of segregation of Ni. This enabled an improved degree of alloying in comparison with the other two studied catalysts, as confirmed by XRD results. Additionally, the enhanced catalytic activity of PdAuNi/C_SBIPA_ could also be associated with the better dispersed multi-metallic particles of smaller average size, confirmed by TEM analysis and the respective EDX elemental maps. Smaller particle size and interparticle distance produced larger surface area and, therefore, were deemed beneficial for catalytic reactions. However, caution must be practiced as Antolini et al. [120] reviewed that decreasing the particle size below 5 nm and interparticle distance below 20 nm adversely impacts the reduction of oxygen, as well as reactions of ethanol and methanol oxidation on Pt surfaces. The situation would be worse for a highly aggregated particle system due to exceedingly smaller interparticle distance. That is a likely reason for the lower PdAuNi/C_SBEG_ performance in the present study. The CVs shown in Figure 6 are also available in specific current density (mA mg_metal_^−1^) in SI (Figure S7).

BOR kinetic parameters are summarized in Table 4 for each catalyst.

**Figure 7:**
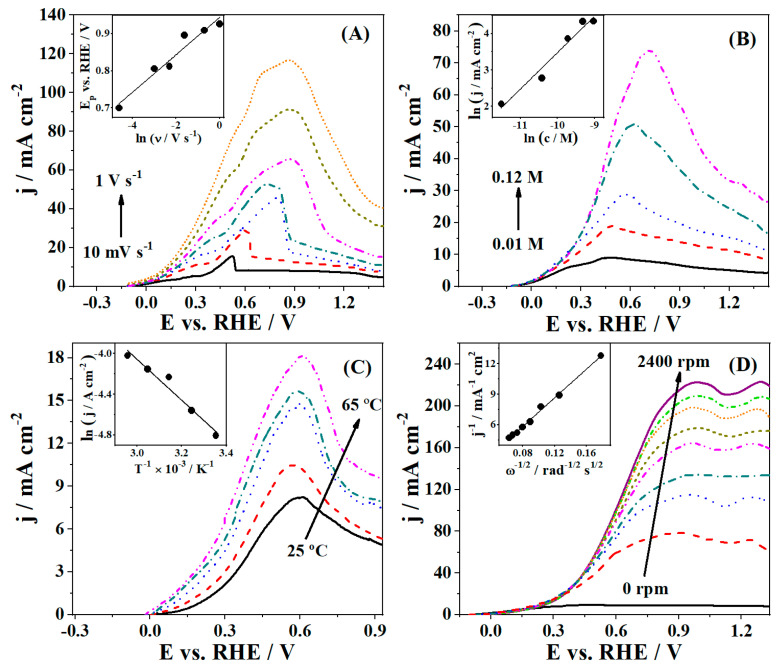
CVs of PdAuNi/C_SBIPA_ at (**A**) different scan rates, ν, and the derived E_p_ vs. ln ν plots (inset), (**B**) different NaBH_4_ concentrations and the derived ln j vs. ln c plots (inset), (**C**) different temperatures and the derived Arrhenius plots (inset), and (**D**) LSVs of PdAuNi/C_SBIPA_ at 10 mV s^−1^ and different rotation rates and the derived Koutecky–Levich plot (inset). The electrolyte solution used was 0.03 M NaBH_4_ + 2 M NaOH, polarization rate was 50 mV s^−1^, with room temperature (25 °C), unless otherwise noted.

For all samples, an upward trend of current density with increasing polarization rate, as well as a small shift in the position of the characteristic BH_4_^−^ oxidation peak to more positive potentials were observed. Figure 7A illustrates this behavior for PdAuNi/C_SBIPA_ as the best material. This allowed for the determination of the charge transfer coefficient (α) values using the expression describing the peak potential dependence on the logarithm of polarization rate (E_p_ vs. ln ν) for irreversible processes [12,121]. The values were found to be relatively similar for all studied catalysts (Table 4), with PdAuNi/C_SBIPA_ showing the highest α value. High values indicate that BOR at examined electrocatalysts was an irreversible and diffusion-controlled process. The obtained values are comparable to the ones reported for catalysts containing Pd, Au, and Ni [12,25,47,79,100,121].

In the case of PdAuNi/C_SBIPA_ (Figure 7B), a significant increase in anodic current densities when recording the CVs starting from 0.01 M to 0.12 M NaBH_4_ concentration was evident. The CVs revealed that the anodic current density value amounted to 8.5 mA cm^−2^ for the lowest concentration (0.01 M), while a value nine times higher was achieved for the highest NaBH_4_ concentration. The observed trend corresponded to enhanced mass transfer, and this behavior agrees with previous reports employing Au-, Pd-, and/or Ni-based electrocatalysts [48,78,79,122,123]. In addition, the oxidation process started at lower potentials but reached its maximum at more positive potential values. The order of reaction (β) values was obtained using ln j vs. ln c plot slope and ranged from 0.91 for PdAuNi/C_SBEG_ to 1.12 for PdAuNi/C_3-step_.

CVs obtained at elevated temperatures also exhibited higher current densities for all studied electrocatalysts. This behavior was expected, since higher temperatures lead simultaneously to a decrease in solution viscosity, resulting in a higher BH_4_^−^ diffusion coefficient, and faster electron transfer kinetics. By constructing the Arrhenius plots [121], depicted in the inset of Figure 7C, and applying the Arrhenius equation, the values of the apparent activation energy, E_a_^app^, were estimated. PdAuNi/C_SBIPA_ showed the lowest value of 16.7 kJ mol^−1^, while the highest value of this parameter was observed for PdAuNi/C_SBEG_ (34 kJ mol^−1^). Similar values were reported for mono- and bimetallic noble metal or/and transition metal-containing materials. For instance, BOR at carbon-supported Pd catalyst (prepared using different synthesis methods and types of carbon support) [53,124] has been shown to proceed with activation energy ranging from as low as 10 kJ mol^−1^ to 26 kJ mol^−1^. Moreover, E_a_^app^ of BOR at a commercially available Pt/C [22] catalyst was reported to have a value of 34 kJ mol^−1^, while alloying with transition metals (Co and Ni) resulted in lower values (25 and 20 kJ mol^−1^, respectively). 

The reaction kinetics were additionally examined using RDE LSVs, and polarization curves were obtained; the corresponding j^−1^ vs. ω^−1/2^ plot for the currents taken at 0.9 V (inset) is shown in Figure 7D. From the slope of j^−1^ vs. ω^−1/2^ plots and using the Koutecky–Levich equation, n values were obtained [125]. The shift of half-wave potential with an increase of the electrode rotation speed confirmed the irreversibility of the reaction.

The n values for PdAuNi/C_3-step_ and PdAuNi/C_SBIPA_ electrocatalysts (7.4 and 7.1, respectively) were determined to nearly match the theoretical value of 8, while significantly lower values were obtained for the other two materials (2.8 and 1.2 for PdAuNi/C_SBEG_ and Pd/C_SBIPA_, respectively). Although in terms of current densities, PdAuNi/C_3-step_ showed significantly lower values than PdAuNi/C_SBIPA_, the highest n value could be explained by Au@Pd (core@shell) structure formation previously identified in the XRD analysis. Such a structure makes the behavior of the catalyst more Au-like due to the core position of those nanoparticles and as such, less active toward the BH_4_^−^ hydrolysis reaction [12,100,126]. The lower faradaic efficiency of PdAuNi/C_SBEG_ and Pd/C_SBIPA_ was most likely a consequence of the higher hydrolysis rates at those two electrocatalysts. Obtained n values for PdAuNi/C_SBEG_ and Pd/C_SBIPA_ are similar to the ones obtained for other Pd-containing materials reported in previous studies. For instance, Martins et al. [54] reported n values of 2 and 5.6 during BOR at Pd nanoparticles with two different types of biobased carbon supports. For other Pd-based catalysts, depending on NaBH_4_ concentration, n values between 2 and 6 [53,118] were obtained, indicating hydrolysis occurrence in parallel with the oxidation of BH_4_^−^. Song et al. [127] recently reported high BOR current densities for a ternary CuPdNi electrode, but at high overpotentials and an n value of 4.9, far from those obtained for the two best catalysts in this work. Alloying Pd with Au resulted in n ~6, as reported by Simões et al. [57], also lower than values than obtained herein for PdAuNi/C_SBEG_ and Pd/C_SBIPA_. Figure 7 is also available in specific current density (Figure S8 of SI).

### 3.3. Fuel Cell Testing

**Figure 8:**
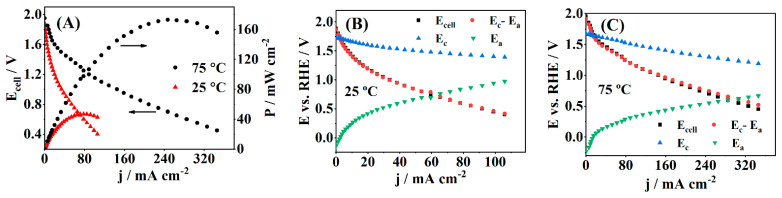
(**A**) Polarization and power density curves at 25 and 75 °C for a DBPFC using a PdAuNi/C_SBIPA_ anode and a Pt cathode. The cathode and anode potentials, their potential difference, and E_cell_ are also represented at (**B**) 25 °C and (**C**) 75 °C.

Figure 8 (available in SI in specific current density, Figure S9) depicts the polarization and power density curves of a single cell with PdAuNi/C_SBIPA_ anode operating at two different temperatures, 25 and 75 °C, along with anode and cathode potentials measured during the cell operation and experimentally measured cell voltage. The cathode potential measured in zero current regime (open-circuit conditions) at 25 °C was almost identical to the equilibrium potential of H_2_O_2_ reduction given in Equation (4) (E^0^ = 1.78 V), suggesting direct H_2_O_2_ reduction. As for the cell operating at elevated temperature (75 °C), this value was slightly less positive, as expected, implying H_2_O_2_ disproportionation was followed by O_2_ reduction to a certain extent [18,21]. On the other hand, the anode potential measured during the experiment was significantly higher than the equilibrium potential for BOR described by Equation (1), implying that the anodic process also involved side reactions, such as oxidation of H and BH_3_OH^−^ generated during spontaneous hydrolysis of BH_4_^−^ [128]. Moreover, from Figure 8B,C, a small difference between cell voltage, E_cell_, and the calculated potential difference of the half-reactions, E_c_–E_a_, could be observed, which revealed minor resistance of system components.

Considering the pH values of electrolytes, the theoretical voltage value of such fuel cells was expected to be close to 3 V. However, the cell polarization curves show an OCV value of ~1.9 V at both temperatures. This fluctuation in OCV value was mainly triggered by the mixed potential occurrence on the anodic side and it agrees with the reports from the literature for DBPFCs studies performed under conditions similar to the ones reported herein [18,125,128]. After the initial voltage decay due to activation losses, cell voltage continued to decrease linearly with current density, while mass transport effects were not evident. The power density value reached its maximum of 47.5 mW cm^−2^ at 0.6 V and 78 mA cm^−2^. As expected, at an elevated temperature of 75 °C, the kinetics of both fuel cell reactions (BOR and HPRR) was faster, and a significant improvement in cell performance was displayed. The maximum value of power density that was obtained at this temperature was almost four times higher than that at 25 °C, amounting to 175 mW cm^−^^2^. This value was exhibited at a cell voltage value of 0.7 V, while current density reached 247 mA cm^−2^. The comparison of the DBPFC results obtained herein with the ones from the literature is shown in Table 5.

PdAuNi/C_SBIPA_ exhibited the best performance among the presented electrocatalysts, even when compared with the typical (and expensive) electrocatalysts exclusively composed of noble metals (e.g., Pd [128], Au/C [129]). It shows that the alloying effect, especially between noble and low-cost metals, is one of the best ways to decrease the price of electrocatalysts while increasing their catalytic activity. In fact, the highest activity of PdAuNi/C_SBIPA_ towards BOR, i.e., the most pronounced synergistic effect, might be due to the complete mixing of three metals by the SBIPA procedure, as indicated by XRD analysis. Furthermore, XRD and XPS analysis indicated the decrease of adsorbate-Pd bond strength that could lead to faster release and subsequent oxidation of the reaction intermediates. Thus, PdAuNi/C_SBIPA_ was demonstrated to be 1.5 and 1.6 times more active (in terms of current density) for BOR than Ni@Pd/PANI [130] and PtNi/C [131] electrocatalysts, which reflects the advantage of using a trimetallic alloy, probably due to the mentioned synergic effect. Carbon-supported Au_x_Ni_y_Cu_z_ trimetallic electrocatalysts [119] exhibited better results than PdAuNi/C_SBIPA_ at high Au amounts (Au_2_NiCu). For the other compositions, PdAuNi/C_SBIPA_ was demonstrated to be a more effective anode, showing that the affinity between the metals is crucial to promote a notable alloying effect.

## 4. Conclusions

PdAuNi carbon-supported trimetallic catalysts were successfully synthesized by three different routes. A PdAuNi/C_SBIPA_ catalyst, synthesized via reduction using a NaBH_4_-2-propanol complex, presented the best alloying effect, while the other two materials showed the presence of segregated Ni species, as confirmed by XRD, TEM, and XPS analyses. The results obtained from CV and LSV measurements revealed that the catalysts performed well towards BOR. The PdAuNi/C_SBIPA_ catalyst exhibited the highest activity, evidenced by the high number of exchanged electrons amounting to 7.1, and the lowest apparent activation energy (ca. 16.7 kJ mol^−1^). Furthermore, DBPFC operating at two different temperatures (25 and 75 °C) employing a PdAuNi/C_SBIPA_ anode showed a maximum power density of 47.5 mW cm^−2^ at 25 °C, with a significant improvement with a temperature increase and peak power density of 175 mW cm^−2^ at 75 °C. Considering the economic benefits of alloying and using support materials, and the results obtained herein, it can be concluded that PdAuNi/C_SBIPA_ is an interesting candidate for the application in DBPFC as anode material.

## Figures and Tables

**Table 1:** Structural and morphological parameters of Pd/C_SBIPA_, PdAuNi/C_SBIPA_, PdAuNi/C_SBEG_, and PdAuNi/C_3-step_.

Catalyst	*2θ*°	FWHM°	*d*-Space (Å)	*a* (Å)	τ_XRD_ (nm)
Pd/C_SBIPA_	39.8	2.19	2.26	3.92	4.3
PdAuNi/C_SBIPA_	39.4	2.47	2.26	3.92	3.8
PdAuNi/C_SBEG_	39.6	2.32	2.26	3.92	4.9
PdAuNi/C_3-step_	38.5	1.11	2.33	4.03	8.9

**Table 2:** ICP-OES metal composition of Pd/C_SBIPA_, PdAuNi/C_SBIPA_, PdAuNi/C_SBEG_, and PdAuNi/C_3-step_.

Catalyst	Pd/wt.%	Au/wt.%	Ni/wt.%
Pd/C_SBIPA_	12	-	-
PdAuNi/C_SBIPA_	5.1	2.6	0.7
PdAuNi/C_SBEG_	4.8	0.8	9.9
PdAuNi/C_3-step_	7.5	5.5	9.3

**Table 3:** Quantitative EDX analysis of PdAuNi/C_SBEG_, PdAuNi/C_3-step_, and PdAuNi/C_SBIPA_ at 10 kV and 20 kV.

	PdAuNi/C_SBEG_				PdAuNi/C_3-step_				PdAuNi/C_SBIPA_			
	10 kV		20 kV		10 kV		20 kV		10 kV		20 kV	
	wt.%	at.%.	wt.%	at.%	wt.%	at.%.	wt.%	at.%	wt.%	at.%.	wt.%	at.%
**C**	63.9	82.7	62.3	80.6	62.6	84.5	61.8	82.8	60.1	83.6	59.3	81.5
**O**	6.67	6.48	8.48	8.25	4.44	4.50	5.42	5.46	4.63	4.84	6.56	6.77
**Ni**	13.4	3.54	12.3	3.25	8.51	2.35	6.74	1.85	10.2	2.90	8.97	2.52
**Pd**	6.03	0.88	6.23	0.91	10.4	1.58	10.7	1.62	11.5	1.80	11.3	1.75
**Au**	3.82	0.30	3.93	0.31	7.96	0.66	8.17	0.67	7.92	0.67	7.56	0.63

**Table 4:** Kinetic parameters for BOR at the four studied catalysts.

Catalyst	α	n	β	E_a_/kJ mol^−^^1^
PdAuNi/C_SBEG_	0.60	2.8	0.91	34.0
PdAuNi/C_SBIPA_	0.75	7.1	1.00	16.7
PdAuNi/C_3-step_	0.73	7.4	1.12	29.9
Pd/C_SBIPA_	0.70	1.2	0.94	28.9

**Table 5:** Comparison of the DBPFC parameters obtained in this work with those reported in similar studies using Pd, Au, Ni, and their alloys.

Anode	Cathode	Fuel	Oxidant	T/°C	OCV/V	P/mW cm^−2^	E_cell, peak_/V	Ref.
PdAuNi/C_SBIPA_	Pt	1 M NaBH_4_ + 4 M NaOH	5 M H_2_O_2_ + 1.5 M HCl	25	1.90	47.5	0.60	This work
				75	1.90	175	0.70	
Au/C	Pt	0.5 M NaBH_4_ + 2 M NaOH	4.5 M H_2_O_2_ + 2 M HCl	20	1.41	8.72	0.42	[119]
AuCu/C	Pt	0.5 M NaBH_4_ + 2 M NaOH	4.5 M H_2_O_2_ + 2 M HCl	20	1.50	31.3	0.49	[119]
AuNi/C	Pt	0.5 M NaBH_4_ + 2 M NaOH	4.5 M H_2_O_2_ + 2 M HCl	20	1.59	40.6	0.57	[119]
Au_1_._5_NiCu/C	Pt	0.5 M NaBH_4_ + 2 M NaOH	4.5 M H_2_O_2_ + 2 M HCl	20	1.62	47.4	0.70	[119]
Au_2_NiCu/C	Pt	0.5 M NaBH_4_ + 2 M NaOH	4.5 M H_2_O_2_ + 2 M HCl	20	1.78	60.5	0.88	[119]
Au/C	Au/C	1 M NaBH_4_ + 3 M NaOH	2 M H_2_O_2_ + 0.5 M H_2_SO_4_	25	1.87	28.2	0.59	[129]
Au_45_Co_55_/C	Au/C	1 M NaBH_4_ + 3 M NaOH	2 M H_2_O_2_ + 0.5 M H_2_SO_4_	25	1.92	66.5	0.77	[129]
Ni@Pd/PANI ^1^	Pt/C	1 M NaBH_4_ + 2 M NaOH	2 M H_2_O_2_ + 1.5 M HCl	60	1.76	120	0.61	[130]
PtNi/C	Pt/C	1 M NaBH_4_ + 2 M NaOH	2 M H_2_O_2_ + 0.5 M H_2_SO_4_	60	1.77	107	0.82	[131]
Pd/MWCNT	Pt/MWCNTs ^2^	5 wt.% NaBH_4_ + 10 wt.% NaOH	5 wt.% H_2_O_2_ + 5 wt.% H_2_PO_4_	25	1.80	119	0.58	[128]

^1^ Ni@Pd (core@shell structure) supported on polyaniline (PANI). ^2^ Pt supported on multiwalled carbon nanotubes (MWCNTs).

## Data Availability

The data presented in this study are available on request from the corresponding author.

## Supplementary Materials

The following are available online at https://www.mdpi.com/article/10.3390/nano11061441/s1, Figure S1: (A) Pd, (B) Au, and (C) Ni elemental maps of PdAuNi/C_3-step_ obtained by EDX spectroscopy at 20 kV, Figure S2: TEM images of Pd/C_SBIPA_ electrocatalyst, Figure S3: TEM image of PdAuNi/C_SBIPA_ electrocatalyst, Figure S4: TEM image of PdAuNi/C_SBEG_ electrocatalyst, Figure S5: TEM images of PdAuNi/C_3-step_ electrocatalyst, Figure S6: XPS survey spectra of Pd/C_SBIPA_, PdAuNi/C_SBIPA_, PdAuNi/C_SBEG_, and PdAuNi/C_3-step_, Figure S7: CVs (third cycle) in 2 M NaOH + 0.03 M NaBH_4_ for (A) PdAuNi/C_SBEG_, (B) PdAuNi/C_SBIPA_, and (C) PdAuNi/C_3-step_ electrocatalysts and (D) comparative CVs of trimetallic and Pd/C_SBIPA_ monometallic catalysts. CVs run at 50 mV s^−1^ and 25 °C, Figure S8: CVs of PdAuNi/C_SBIPA_ at (A) different scan rates, ν, and the derived E_p_ vs. ln ν plots (inset), (B) different NaBH_4_ concentrations and the derived ln j vs. ln c plots (inset), (C) different temperatures and the derived Arrhenius plots (inset), and (D) LSVs of PdAuNi/C_SBIPA_ at 10 mV s^−1^ and different rotation rates and the derived Koutecky–Levich plot (inset). The electrolyte solution used was 0.03 M NaBH_4_ + 2 M NaOH, polarization rate was 50 mV s^−1^, and room temperature (25 °C), unless otherwise noted, Figure S9: (A) Polarization and power density curves at 25 and 75 °C for a DBPFC using a PdAuNi/C_SBIPA_ anode and a Pt cathode. The cathode and anode potentials, their potential difference, and E_cell_ are also represented at (B) 25 °C and (C) 75 °C., Table S1: XPS elemental peaks of Pd 3d, Au 4f, and Ni 2p in Pd/C_SBIPA_, PdAuNi/C_SBIPA_, PdAuNi/C_SBEG_, and PdAuNi/C_3-step_.
