# Supplementary material for: Carbon-Supported Trimetallic Catalysts (PdAuNi/C) for Borohydride Oxidation Reaction
Source: Nanomaterials (Basel). 2021 May 29;11(6):1441. doi: 10.3390/nano11061441 (PMC8228588; doi:10.3390/nano11061441)
Supplement: Supplementary file 1 [file nanomaterials-11-01441-s001.zip › nanomaterials-1229207-SI.pdf]

nanomaterials

MDPI

Supplementary Materials

Carbon-Supported Trimetallic Catalysts (PdAuNi/C) for Borohydride Oxidation Reaction

Ahmed M. A. ElSheikh [1,2,†], Gordana Backović [3,†], Raisa C. P. Oliveira [3], César A. C. Sequeira [3], James McGregor [2], Biljana Šljukić [3] and Diogo M. F. Santos [3,*]

[1] Mechanical Engineering Department, South Valley University, AlShobaan AlMoslemeen Street, Qena 83521, Egypt; ahmed.elsheikh@eng.svu.edu.eg
[2] Department of Chemical and Biological Engineering, University of Sheffield, Sheffield S1 3JD, UK; james.mcgregor@sheffield.ac.uk
[3] Center of Physics and Engineering of Advanced Materials (CeFEMA), Instituto Superior Técnico, Universidade de Lisboa, 1049-001 Lisboa, Portugal; gordanabackovic@tecnico.ulisboa.pt (G.B.) raisa.oliveira@tecnico.ulisboa.pt (R.C.P.O.); cesarsequeira@tecnico.ulisboa.pt (C.A.C.S.); biljana.paunkovic@tecnico.ulisboa.pt (B.Š.)
* Correspondence: diogosantos@tecnico.ulisboa.pt; Tel.: +351-218417765
† Equally contributed.

1. XRD Calculation

Structural parameters: interplanar spacing and lattice constant have been estimated according to Bragg's law (Equations S1 and S2),

$$n\lambda = 2d\sin(\theta) \qquad \text{(S1)}$$

$$a = d_{hkl} \times (h^2 + k^2 + l^2)^{1/2} \qquad \text{(S2)}$$

where n=1 and λ (wavelength of Cu K_α) = 0.154 nm, θ is the half of diffraction angle (degrees), d_{hkl} is the interplanar distance (nm). The values of the *d*-spacing and lattice constant of the four studied catalysts are listed in Table 1 of the manuscript (MS). The particle size is estimated according to the Scherrer equation (Equation S3),

$$\tau = k\lambda \times [\beta\cos(\theta)]^{-1} \qquad \text{(S3)}$$

where τ is the particle size (nm), k is a dimensionless constant (0.94), and β (or FWHMº in Table 1 of the MS) is the full width at half maximum height (in radians).

2. EDX Data of PdAuNi/C3-step

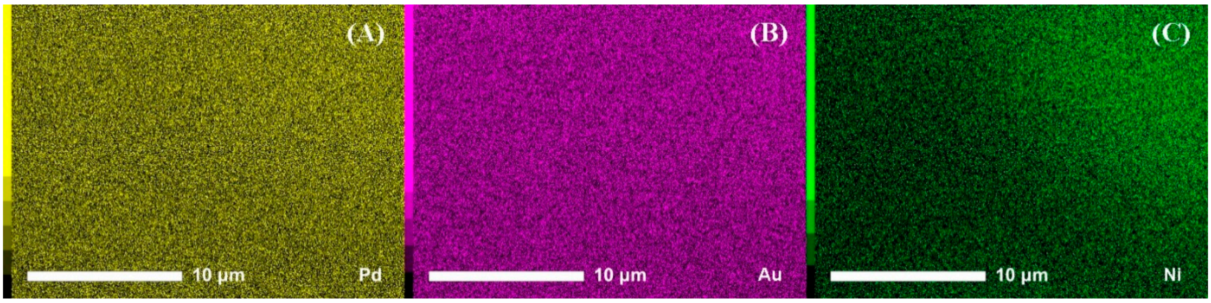

Figure S1. (**A**) Pd, (**B**) Au, and (**C**) Ni elemental maps of PdAuNi/C3-step obtained by EDX spectroscopy at 20 kV.

3. TEM Micrographs

3.1. Pd/CSBIPA

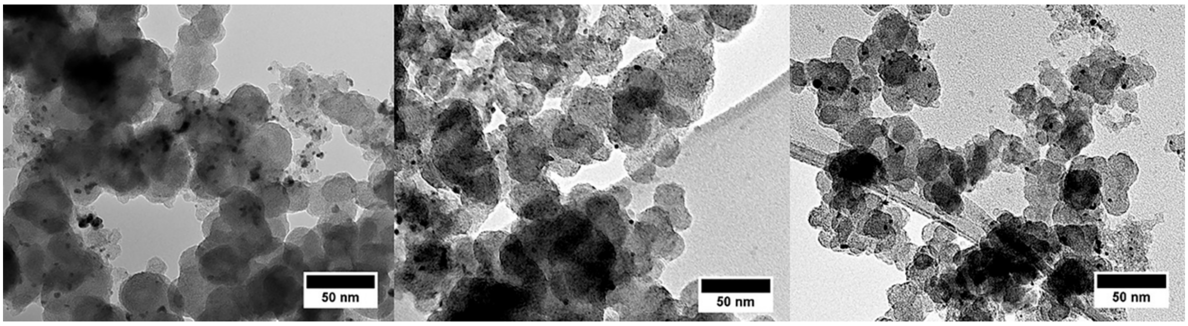

Figure S2. TEM images of Pd/CSBIPA electrocatalyst.

3.2. PdAuNi/CSBIPA

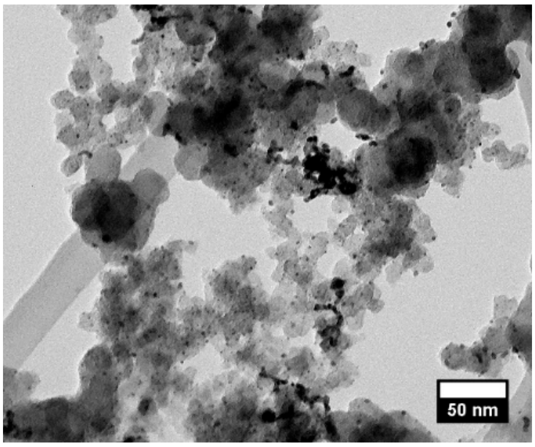

Figure S3. TEM image of PdAuNi/CSBIPA electrocatalyst.

3.3. PdAuNi/C$_{SBEG}$

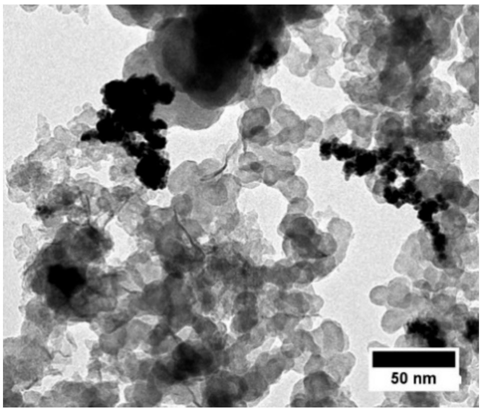

Figure S4. TEM image of PdAuNi/C$_{SBEG}$ electrocatalyst.

3.4. PdAuNi/C$_{3\text{-}step}$

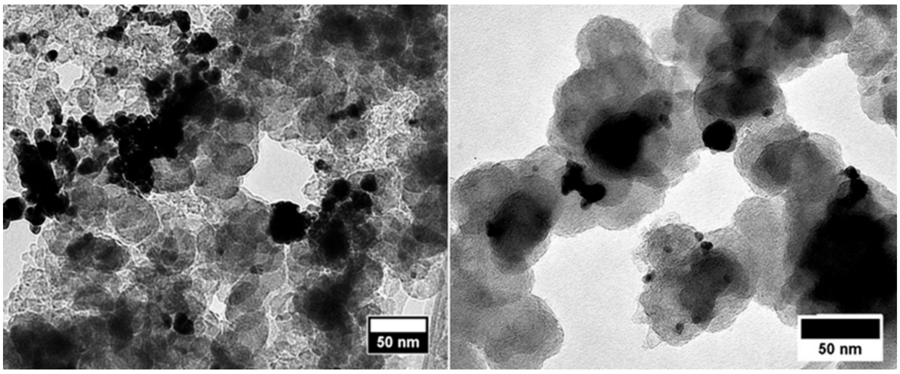

Figure S5. TEM images of PdAuNi/C$_{3\text{-}step}$ electrocatalyst.

4. XPS Data

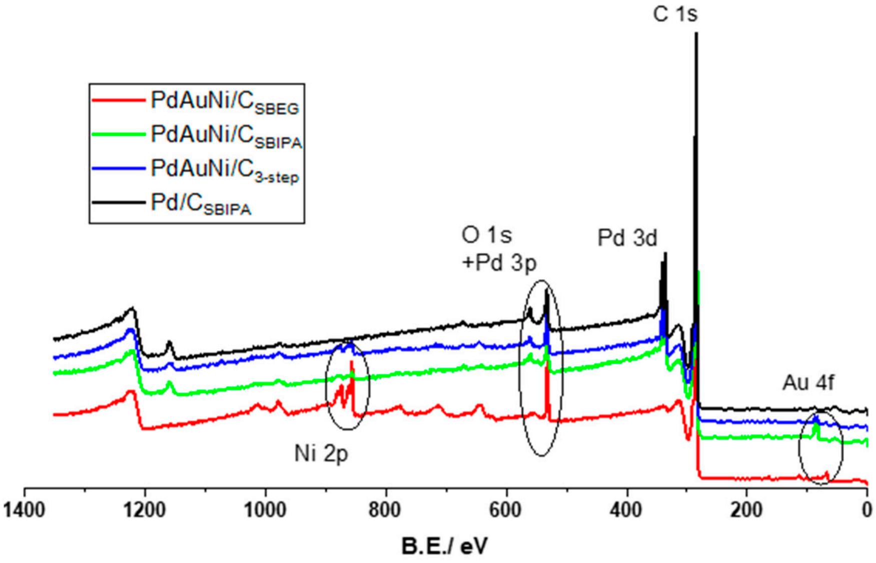

Figure S6. XPS survey spectra of Pd/C$_{SBIPA}$, PdAuNi/C$_{SBIPA}$, PdAuNi/C$_{SBEG}$, and PdAuNi/C$_{3\text{-}step}$.

Table S1. XPS elemental peaks of Pd 3d, Au 4f, and Ni 2p in Pd/C_{SBIPA}, PdAuNi/C_{SBIPA}, PdAuNi/C_{SBEG}, and PdAuNi/C_{3-step}.

Catalyst	Pd 3d	Au 4f	Ni 2p
Pd/C$_{SBIPA}$	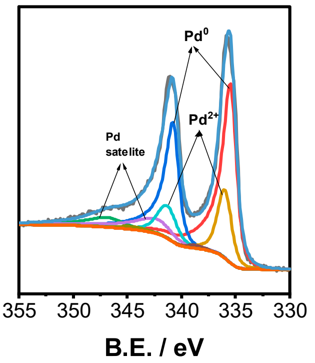		
PdAuNi/C$_{SBIPA}$	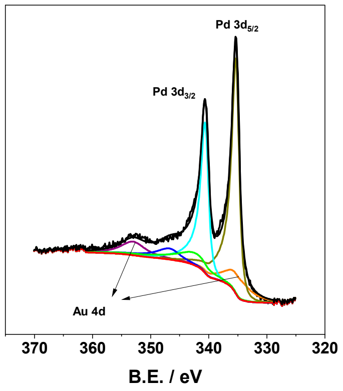	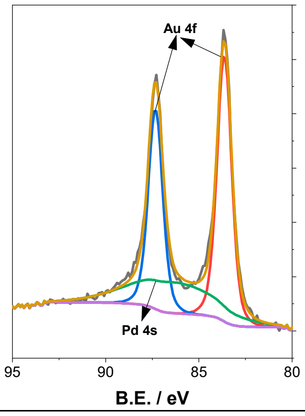	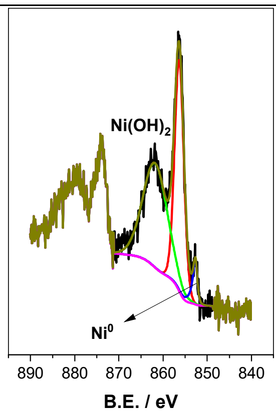
PdAuNi/C$_{SBEG}$	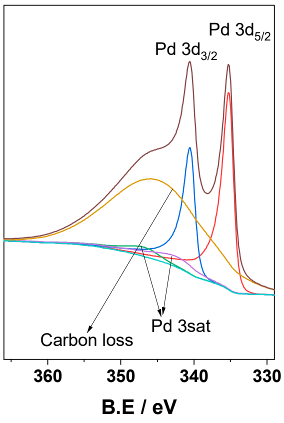	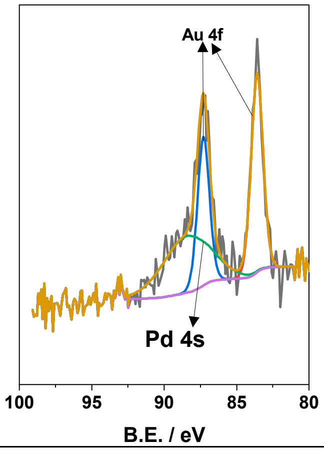	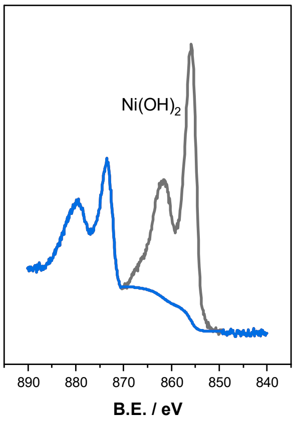
PdAuNi/C$_{3\text{-}step}$	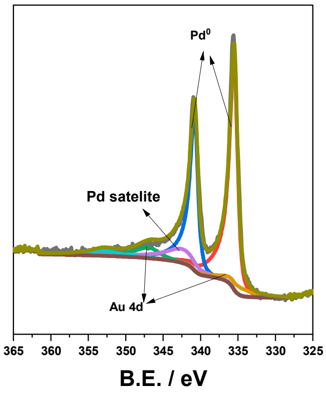	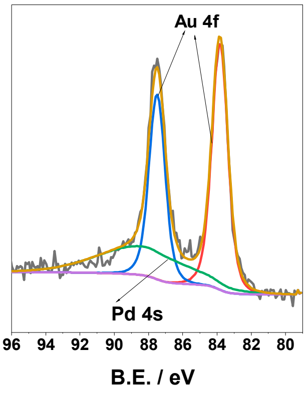	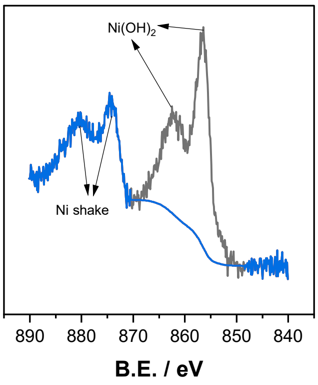

5. Determination of the Number of Exchanged Electrons by Koutecky-Levich Equation

The determination of the number of exchanged electrons was done through RDE studies (Figure 7D of the manuscript and its inset), by applying the Koutecky-Levich equation (Equation S4),

$$\frac{1}{j}=\frac{1}{j_d}+\frac{1}{j_k}=\frac{1}{0.62nFD^{2/3}\upsilon^{-1/6}C\omega^{1/2}}+\frac{1}{j_k} \quad \text{(S4)}$$

where j_d and j_k are the diffusion-limited and kinetic current densities (mA cm^{-2}), n is the number of exchanged electrons, F is the Faraday constant (96485 C), D is the diffusion coefficient of BH_4^- (cm^2 s^{-1}), υ is the kinematic viscosity of the solution (cm^2 s^{-1}), C is the BH_4^- concentration (mol cm^{-3}), and ω is the rotation rate (rad s^{-1}).

6. Figures 6, 7 and 8 of the Manuscript, Involving the Electrochemical Studies and Fuel Cell Tests, with the Currents Given as Specific Current Densities

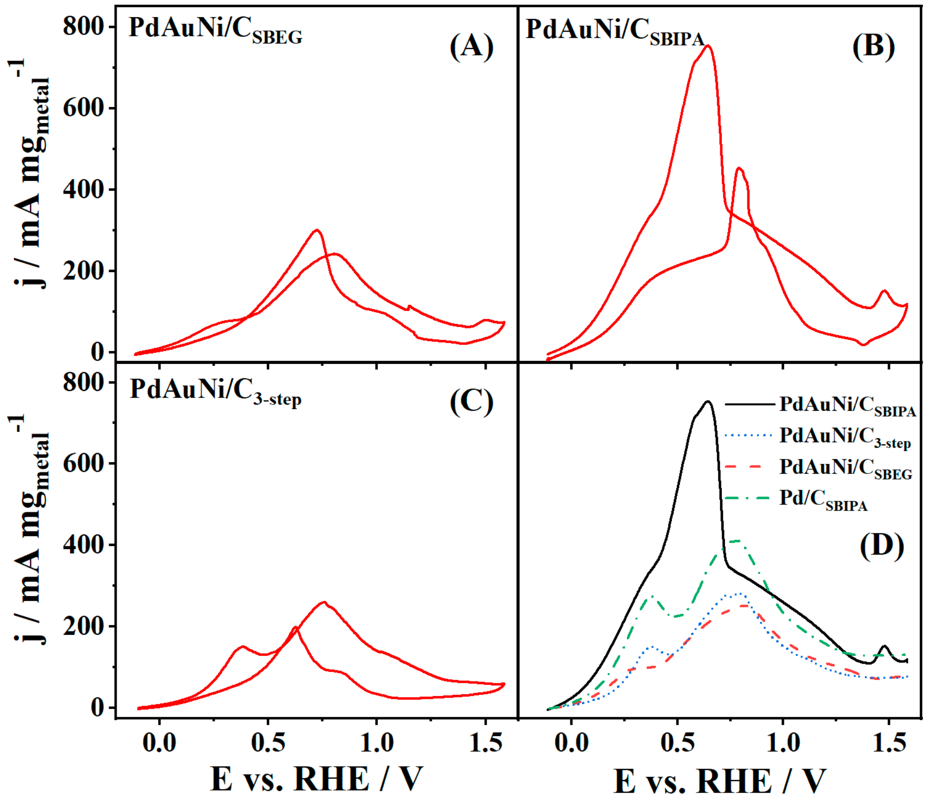

Figure S7. CVs (third cycle) in 2 M NaOH + 0.03 M $NaBH_4$ for (**A**) PdAuNi/C$_{SBEG}$, (**B**) PdAuNi/C$_{SBIPA}$, and (**C**) PdAuNi/C$_{3-step}$ electrocatalysts and (**D**) comparative CVs of trimetallic and Pd/C$_{SBIPA}$ monometallic catalysts. CVs run at 50 mV s^{-1} and 25 ºC.

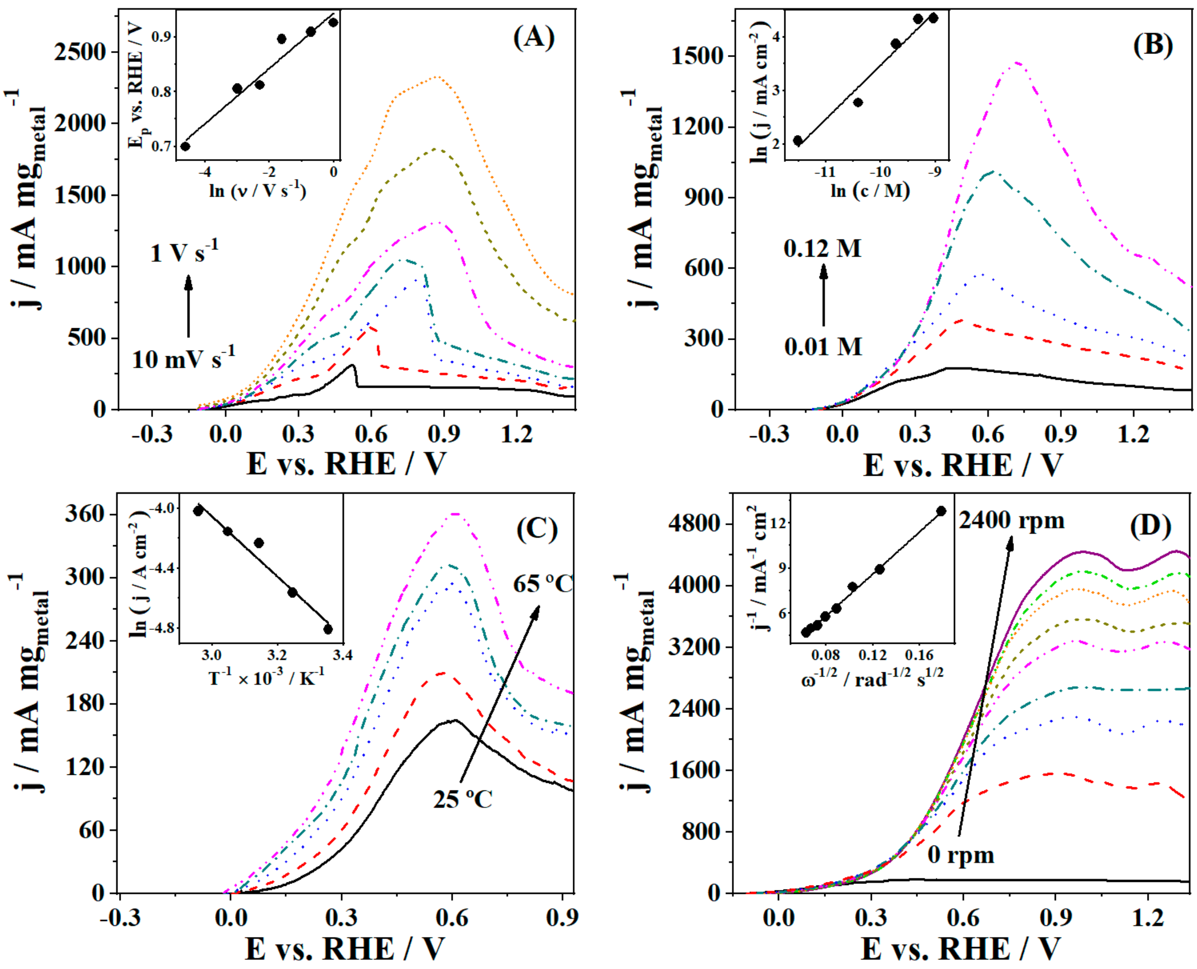

Figure S8. CVs of PdAuNi/C$_{SBIPA}$ at (**A**) different scan rates, ν, and the derived E_p vs. ln ν plots (inset), (**B**) different $NaBH_4$ concentrations and the derived ln j vs. ln c plots (inset), (**C**) different temperatures and the derived Arrhenius plots (inset), and (**D**) LSVs of PdAuNi/C$_{SBIPA}$ at 10 mV s^{-1} and different rotation rates and the derived Koutecky–Levich plot (inset). The electrolyte solution used was 0.03 M $NaBH_4$ + 2 M NaOH, polarization rate was 50 mV s^{-1}, and room temperature (25 ºC), unless otherwise noted.

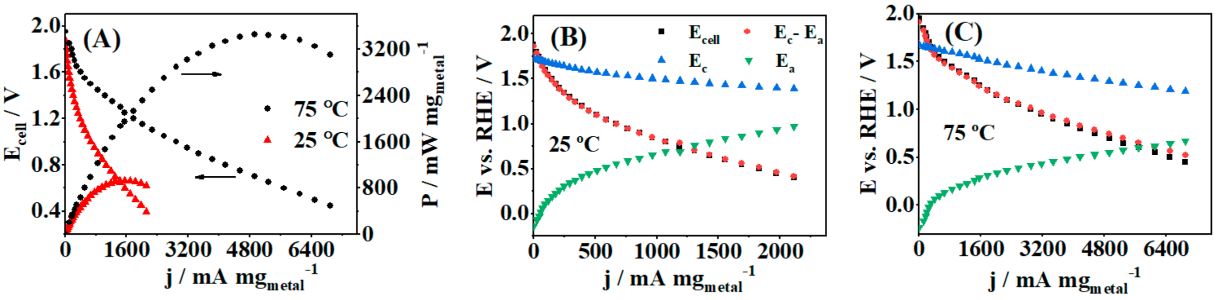

Figure S9. (**A**) Polarization and power density curves at 25 and 75 ºC for a DBPFC using a PdAuNi/C$_{SBIPA}$ anode and a Pt cathode. The cathode and anode potentials, their potential difference, and E_{cell} are also represented at (**B**) 25 ºC and (**C**) 75 ºC.
